# Pseudomyxoma peritonei leading to “jelly belly” abdomen: a case report and review of the literature

**DOI:** 10.1186/s13256-024-04612-1

**Published:** 2024-06-28

**Authors:** Priyanka Garg, Nikhil Garg, Sameer Peer, Deepika Chholak, Manjit Kaur

**Affiliations:** 1 Department of Obstetrics and Gynecology, All India Institute of Medical Sciences, Bathinda, Punjab 151001 India; 2 Department of Surgical Oncology, All India Institute of Medical Sciences, Bathinda, Punjab 151001 India; 3 Department of Radiology, All India Institute of Medical Sciences, Bathinda, Punjab 151001 India; 4 Department of Pathology, All India Institute of Medical Sciences, Bathinda, Punjab 151001 India

**Keywords:** Pseudomyxoma peritonei, Peritoneal neoplasms, Mucinous, Adenocarcinoma, Cytoreductive surgical procedures

## Abstract

**Background:**

Pseudomyxoma peritonei is an infrequent condition with a global annual incidence of only one to two cases per million people. Mucinous neoplasms, widespread intraperitoneal implants, and mucinous ascites characterize it. Currently, most clinicians misdiagnose this condition, which leads to delayed management.

**Case presentation:**

A 44-year-old North Indian female presented with a 1.5-month history of an abdominal lump. Physical examination revealed a sizeable abdominopelvic mass at 36 weeks. Contrast-enhanced computed tomography showed a massive multiloculated right ovarian cystic mass measuring 28 × 23 × 13 cm with mild ascites and elevated carcinoembryonic antigen levels (113.75 ng/ml). A provisional diagnosis of ovarian mucinous neoplasm was made, for which the patient underwent laparotomy. Intraoperatively, there were gross mucinous ascites, along with a large, circumscribed, ruptured right ovarian tumor filled with gelatinous material. The appendicular lump was also filled with mucinous material along with the omentum, ascending colon, right lateral aspect of the rectum, splenic surface, and small bowel mesentery. Cytoreductive surgery was performed along with an oncosurgeon, including total abdominal hysterectomy with bilateral salpingoophorectomy, omentectomy, right hemicolectomy, lower anterior resection, ileo-transverse stapled anastomosis with proximal ileal loop diversion stoma, excision of multiple peritoneal gelatinous implants, and peritoneal lavage. Histopathology and immunohistochemistry confirmed the presence of intestinal-type mucinous carcinoma. Postoperatively, the patient was given six cycles of chemotherapy. She tolerated it without any specific morbidity and had an uneventful recovery. Postoperative follow-up at 15 months revealed normal tumor marker levels and abdominal computed tomography findings and no signs suggestive of local recurrence or distal metastases.

**Conclusions:**

Pseudomyxoma peritonei is a rare disease that is frequently misdiagnosed in the preoperative phase. Therefore, radiologists and clinicians should maintain a high index of suspicion for accurate diagnosis and multidisciplinary management.

## Introduction

Pseudomyxoma peritonei (PMP) is a rare condition caused by primary mucinous tumors arising from different locations, usually the appendix or ovary; other rare sites include the gallbladder, stomach, colorectum, fallopian tube, urachus, lung, and breast. It is characterized by mucin production in the abdominal cavity and, if left untreated, can compress adjacent vital organs. The term “PMP” was first given by Werth in 1884, and the current incidence is estimated to be one to two cases per million per year [1]. With a mean incidence at the age of 53 years, PMP is more prevalent in women (male-to-female ratio, 9:11) [2]. The clinical features are often nonspecific and may manifest as an inexplicable increase in abdominal girth, abdominal pain, ascites, unilateral or bilateral ovarian tumors, bowel obstruction, or appendicitis-like symptoms. The initial diagnostic modality is ultrasonography (USG), followed by computed tomography (CT) or magnetic resonance imaging (MRI) of the abdomen/pelvis. Most of the time, however, the diagnosis is often missed during preoperative evaluation, and the tumor is discovered accidentally during surgery [3]. The serum tumor markers cancer antigen (CA) 19–9 and carcinoembryonic antigen (CEA) aid in diagnosis and have prognostic value [2]. The definitive management consists of complete cytoreductive surgery (CRS) to achieve macroscopic tumor removal, followed by intraperitoneal or systemic chemotherapy to treat microscopic residual disease [4]. However, no standard guidelines are available for the treatment of these patients. In March 2018, the National Comprehensive Cancer Network (NCCN) published a guideline stating that, in recent research, completion of CRS was associated with improving patients’ overall survival, while hyperthermic intraoperative peritoneal chemotherapy (HIPEC) did not [5]. Hence, long-term survival and complete cure have limited expectations [5]. Thus, more robust data need to be generated to provide a less harmful therapeutic approach in an individualized manner along with palliative therapy for those who are ineligible candidates for surgery. PMP should be managed with a multidisciplinary team approach involving a gynecologist, oncosurgeon, radiologist, pathologist, and medical oncologist. We present one such rare case of PMP in a female who was missed on imaging and diagnosed intraoperatively. She was successfully managed with surgical debulking plus chemotherapy. To the best of our knowledge, this is one of the few case reports published in India that has highlighted such surgeries and generated awareness in favor of them.

## Case presentation

A 44-year-old North Indian (para 2, live 2) patient with a previous cesarean section came to the gynecology out-patient department (OPD) with complaints of a lump in the abdomen for the last 1.5 months. Initially, the size of the lump reached the umbilicus, which suddenly increased to the xiphisternum. There was associated abdominal discomfort but no nausea, vomiting, or fever. Her previous medical and family history was unremarkable. On general examination, the patient was, on average, built and afebrile, with no evidence of anemia, jaundice, cyanosis, lymphadenopathy, clubbing, or weight loss. She had normal bladder and bowel function. Her menstrual cycles were regular with normal flow. An abdominal examination revealed a large abdominopelvic mass corresponding to the epigastrium for up to 36 weeks. It was cystic in consistency with a smooth surface, non-tender, and slightly mobile in the transverse plane. The lack of shifting dullness negated the presence of ascites. A per-vaginal examination confirmed an abdominopelvic mass of 36 weeks in size with a normal external vulva and cervix. The uterus could not be appreciated separately. Ultrasound revealed a large abdominopelvic mass with internal septations reaching the epigastrium. The uterus was normal in size, with mild ascites and bilateral adnexa obscured by the mass. On further evaluation, contrast-enhanced computed tomography (CECT) was performed to characterize the mass, which showed a large multiloculated abdominopelvic cystic mass measuring 28 × 23 × 13 cm with the right ovary not seen separately, suggesting a right ovarian origin.

**Fig. 1:**
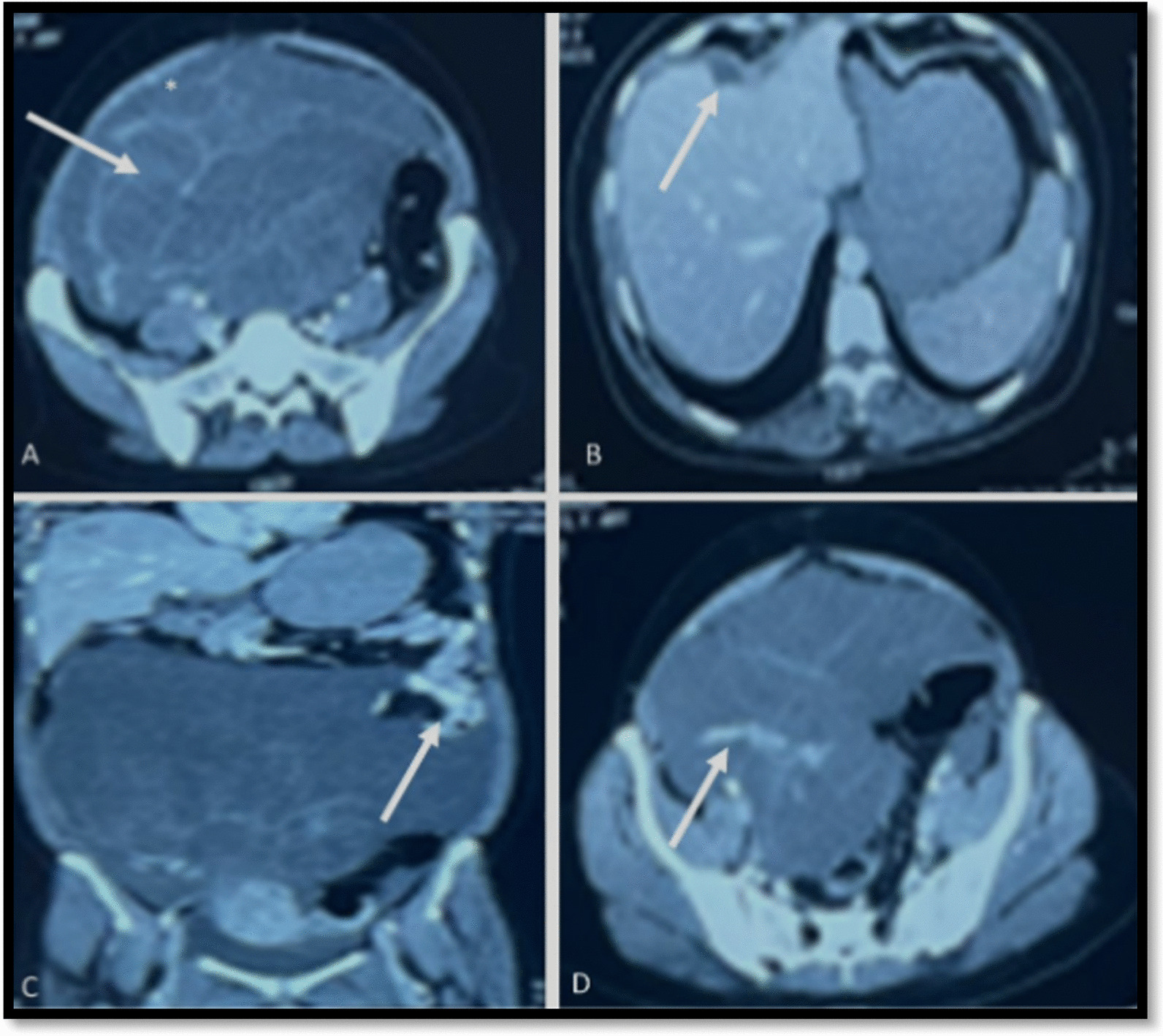
Axial contrast-enhanced computed tomography images of the abdomen and pelvis. The arrows point toward (**A**) a multiseptated abdominopelvic mass lesion with enhancing septations and relatively hyperdense ascites surrounding the mass; **B** scalloping of the anterior margin of the liver (arrow) caused by the ascitic fluid; **C** displacement of the small bowel loops (arrow) by the abdominopelvic mass and ascitic fluid, suggesting a mass effect in coronal reconstruction; and **D** enhancing appendix engulfed within the abdominopelvic mass (arrow) in axial contrast-enhanced computed tomography

The uterus and left ovary were normal and displaced by the mass. Mild ascites was present. Multiple thick enhancing septa were also observed within the mass with no solid component **(**Fig. 1**).** Her CA 125 and CA 19–9 levels were normal (35.4 U/ml and 18.85 U/ml, respectively). However, her CEA level was elevated to 113.75 ng/ml (normal range 0–3 ng/ml). A provisional diagnosis of a right ovarian mucinous tumor was made, and the patient underwent exploratory laparotomy after providing informed consent. Intraoperative findings revealed gelatinous material filling the entire abdominal cavity and pelvis. A large, circumscribed, ruptured tumor arose from the right ovary filled with gelatinous material.

**Fig. 2:**
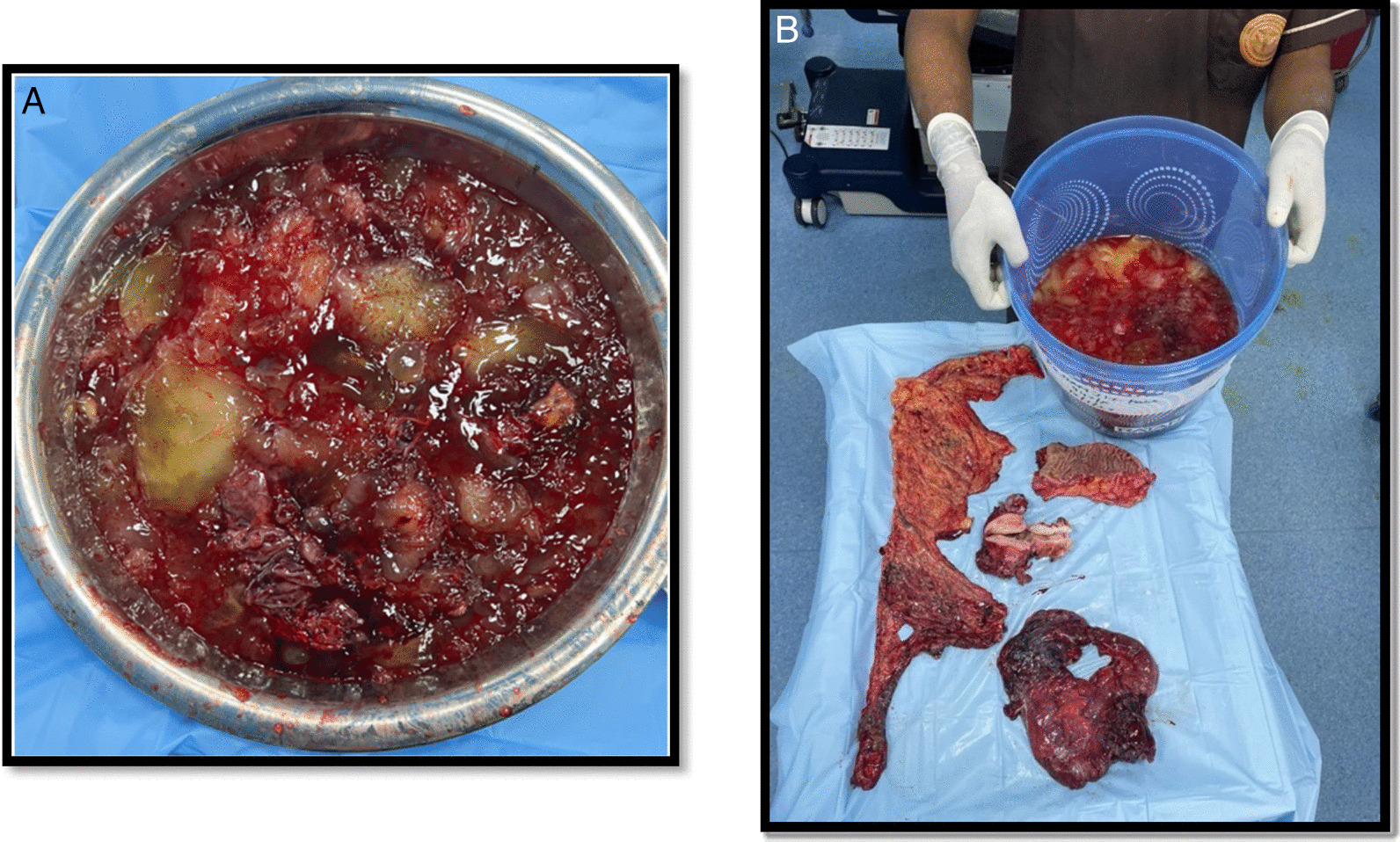
**A** Postoperative images showing yellow gelatinous material and **B** total abdominal hysterectomy with bilateral salpingoophorectomy, omentectomy, and right hemicolectomy with lower anterior resection

**Fig. 3:**
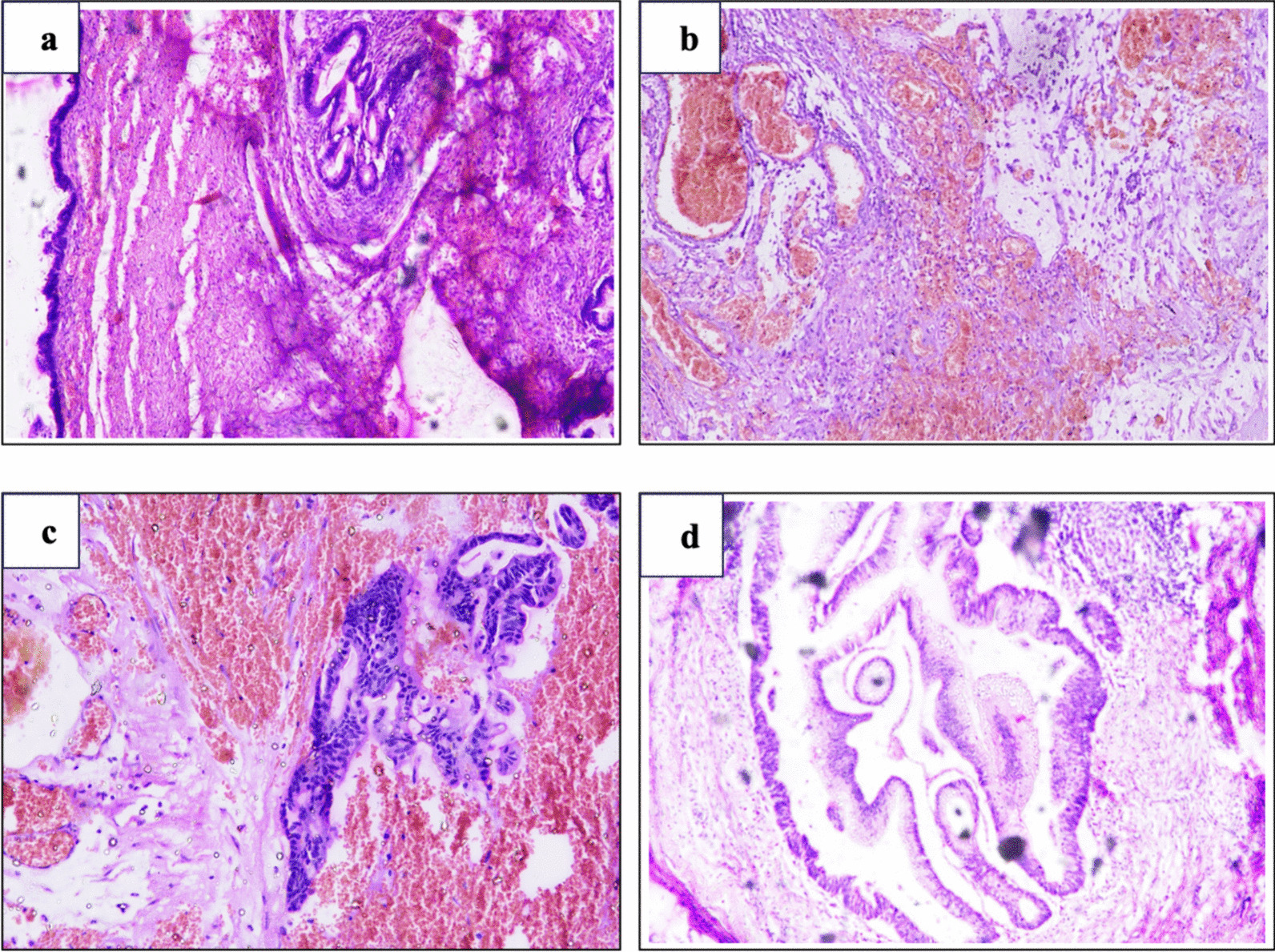
Low-power view of a section of **A** ovary **B**, **C** omentum, and **D** colon showing mucin deposits containing tumor cells (hematoxylin and eosin, 10×)

There were mucinous deposits on the anterior surface of the normal-sized uterus and left ovary. The appendicular lump was also filled with gelatinous material. Multiple gelatinous nodules were present on the omentum, along the ascending colon, right lateral aspect of the rectum, splenic surface, and small bowel mesentery. The oncosurgeon was called intraoperatively, and we performed CRS, including total abdominal hysterectomy with bilateral salpingoophorectomy, total omentectomy, right hemicolectomy, lower anterior resection, ileo-transverse stapled anastomosis with proximal ileal loop diversion stoma, excision of multifocal peritoneal mucinous implants, and peritoneal lavage (Fig. 2). Surgical specimens were subjected to histopathological and immunohistochemical (IHC) examination. Microscopically, the tumor cells were arranged as back-to-back glands, papillae, and cysts lined by mucinous columnar epithelium (intestinal type), with oval to elongated nuclei, a high nucleocytoplasmic ratio, and vesicular nuclei. Stromal infiltration by tumors was noted in the form of nests, irregularly shaped glands, and cords of tumor cells. Abundant extravasated mucin was also observed, revealing mucinous carcinoma of the intestinal type (Fig. 3). On IHC, the tumor cells were CK20+ , CDX2+ , and CK7−, which indicate mucinous neoplasms of the intestinal type. The patient was given six cycles of chemotherapy with carboplatin and paclitaxel. She had an uneventful recovery (as of publication) for 15 months following the operation, with normal tumor marker levels and abdominal CT findings and no signs of local recurrence or distal metastases.

## Discussion

PMP is a poorly understood clinical condition characterized by intra-abdominal mucin accumulation secondary to the proliferation of malignant mucin-secreting cells on the peritoneal surface. In contrast to most malignancies, it rarely spreads via the hematogenous or lymphatic system [6]. Hence, there is nowhere for extracellular mucin to drain, which accumulates significantly in the abdominal cavity and causes the so-called jelly belly abdomen. It was first described by Carl Von Rokitansky in 1842, but the term was used in 1884 in association with a mucinous carcinoma of the ovary [7]. Later, in 1901, Franckel described PMP as an appendiceal cystic tumor [8]. The origin of PMP is still debatable. Most authors agree that PMP develops mainly in the appendix in men, and mounting evidence supports a similar genesis in women. Worldwide, 30–50% of women have simultaneous ovarian and appendiceal mucinous tumors [9]. However, immunohistochemical and molecular genetic approaches support the concept that most ovarian tumors in women are metastatic to a perforated appendiceal mucinous tumor. This notion is supported by our case, in which histological examination of the oophorectomy specimen revealed a pattern consistent with intestinal/appendicular origin.

The diagnosis of PMP is often challenging for clinicians, as the majority of patients remain asymptomatic or present with nonspecific clinical features, such as pain in the abdomen, nausea, vomiting, abdominal distention (as seen in our case), or mass. Approximately one-third of female patients present with an ovarian tumor, as observed in our case [10]. The excessive accumulation of mucin in the peritoneal cavity can compress adjacent structures, leading to bowel obstruction and malnutrition. Touloumis Z *et al*. reported a case of PMP in a 71-year-old patient who presented with intermittent diarrhea without any other symptoms [6].

Imaging studies are also not very helpful in reaching the diagnosis. USG is the initial investigation that may show highly echogenic ascitic fluid with immobile echogenic septations and a marked laminated appearance (onion-skin effect), reflecting the concentric layering of mucin, typical of gelatinous material [4]. The gold standard for imaging is CECT. The usual appearance includes areas of low attenuation, with islands of more significant attenuation resulting from solid elements within the mucinous material. Classically, “scalloping” of visceral surfaces, particularly of the liver and spleen, distinguishes mucinous from fluid ascites [5]. However, most cases are discovered incidentally during laparoscopy or laparotomy [11]. Mathur S *et al*. reported the case of a 27-year-old patient who was incidentally diagnosed with disseminated PMP at the time of a cesarean section [3]. According to the study conducted by Jarvinen and Lepisto, only 28% of patients underwent primary surgery for suspected PMP. The most frequent preoperative diagnosis is appendicitis, gynecological cancer, or ovarian tumor, as in our case, where the diagnosis of PMP was missed on imaging [3].

Tumor markers such as CEA, CA 19–9, and CA-125 are associated with PMP. CEA is a beneficial prognostic marker during diagnosis and postoperative surveillance [2]. Preoperative elevation of serum markers indicates aggressive disease and a high chance of recurrence. Canbay *et al*. noted that preoperative CEA levels can predict disease severity, surgical success, and overall survival in patients treated with CRS and hyperthermic intraoperative peritoneal chemotherapy (HIPEC) [12].

The definitive diagnosis mandates the presence of a mucinous neoplastic cells/epithelium and diffuse intra-abdominal mucin [13]. Patients without epithelium were considered to have mucinous ascites. Epithelial glandular cells must be present in the mucin pool on histopathology to confirm the diagnosis of PMP. The biopsy of our patient concurred. The Peritoneal Surface Oncology Group International (PSOGI) pathologic categorization for PMP now uses distinct nomenclature for treatment selection, as presented in Table 1.

**Table 1:** Histological classification of PMP [14]

Pathological lesion	Criteria
Acellular mucin	Mucin within the peritoneal cavity without neoplastic epithelial cells
Low-grade mucinous carcinoma peritonei (DPAM)	Epithelial component typically scanty, minimal cytologic atypia, strips or gland-like structures, or small cell clusters
High-grade mucinous carcinoma peritonei (PMCA)	Relatively more cellular, cribriform growth pattern, high-grade cytologic atypia, and numerous mitoses
High-grade mucinous carcinoma peritonei with signet rings cells (PMCA-S)	Any lesion with a signet ring cell component, that is, round cells with intracytoplasmic mucin pushing the nucleus against the cell membrane

DPAM, disseminated peritoneal adenomucinosis; PMCA, peritoneal mucinous carcinomatosis; PMCA-S, mucinous carcinoma peritonei with signet rings cells

Appendiceal adenocarcinomas are classified into three histological categories. The most frequent form, mucinous, produces a large amount of mucin. The less prevalent intestinal or colonic form (our case) closely resembles colon adenocarcinomas. Signet ring cell adenocarcinoma is a rare and aggressive cancer with a poor prognosis [2, 15].

Traditionally, the management of PMP includes repeated drainage of gelatinous ascites or serial debulking surgeries involving the removal of the primary tumor and mucinous ascites. However, repeat surgical procedures have become increasingly difficult due to adhesions and fibrous scar tissue formation [16]. These patients eventually die due to severe malnutrition, intestinal obstruction, or surgical complications. Recent studies support the idea that combined cytoreductive surgery with intraperitoneal chemotherapy (IPEC) should be the standard of care because it has improved survival rates. The 5-year survival rate of patients with CRS and IPEC for low-grade disease is 60–100%, whereas for high-grade disease, it is 0–60% [16]. CRS aims to eliminate as many macroscopic illnesses as possible. Sugarbaker’s protocol is commonly used for peritonectomy procedures, which may include anterior parietal peritonectomy, total omentectomy, splenectomy, distal pancreatectomy, right and left subphrenic peritonectomy, Glisson’s capsule removal, pelvic peritonectomy, cholecystectomy, and visceral resections such as rectosigmoidectomy, right colectomy, total abdominal colectomy, and hysterectomy with bilateral salpingo-oophorectomy and small bowel resection [11]. This system is supplemented with IPEC, which targets microscopic residual disease and free neoplastic cells in the peritoneal cavity. Heated/hyperthermic IPEC (HIPEC) can be utilized intraoperatively or early postoperatively [early postoperative intraperitoneal chemotherapy (EPIC)], comparable to peritoneal dialysis. According to a study by Sorensen *et al*., there appears to be no difference in survival rates between the two IPEC procedures [17]. The treatment plan for PMP should aim for full cytoreduction to avoid recurrence or progression. This method is currently being used in numerous centers worldwide, with encouraging results, and appears to be gaining traction compared with traditional serial debulking. However, not all situations are appropriate for this strategy; every center or surgeon is not equipped to perform IPEC, and sometimes, patients might be medically unfit to receive these treatments safely. A randomized study by Verwaal revealed that patients involved in six or more abdominal cavity regions achieved minimal improvement in survival following CRS and intraoperative HIPEC [18]. Therefore, debulking surgeries alone are still being performed and have an overall survival rate of approximately 50% [19]. Additionally, currently, there is no consensus regarding the role of CRS and HIPEC treatment for more aggressive histological variations in PMP. Consequently, they provide evidence favoring systemic chemotherapy as the standard of care for such patients [20]. Per the retrospective research by Shapiro J *et al*., patients considered inadequate candidates for CRS and/or HIPEC therapy benefited from extended disease remission of 7.6 months with contemporary systemic chemotherapy [21]. In another published review of consensus statements and guidelines by the PSOGI expert panel, neoadjuvant or adjuvant systemic chemotherapy can be considered in patients with low-grade PMP and high-grade PMP with signet ring cells. When required, fluoropyrimidine in conjunction with an alkylating drug (such as oxaliplatin) is advised [22]. Currently, there is no standard systemic chemotherapy regimen, and we are unaware of any prospective clinical trials in this patient cohort that involve contemporary systemic chemotherapy and/or biologic therapy. The patient in the present study was treated with CRS, which consisted of hysterectomy with bilateral salpingo‑oophorectomy, omentectomy, right hemicolectomy, lower anterior resection, ileo-transverse stapled anastomosis with proximal ileal loop diversion stoma, excision of multiple peritoneal mucinous implants, and peritoneal lavage. Since HIPEC was not available in our center, the patient was given six cycles of postoperative systemic chemotherapy, and she has been tumor-free to date.

Finally, PMP may relapse despite CRS and chemotherapy (HIPEC, IPEC, or systemic), mainly if the disease is diagnosed at an advanced stage. Tumor marker assessment and routine postoperative CT scans should be utilized to monitor any recurrence.

## Conclusion

PMP is a rare condition that causes significant morbidity and mortality if left untreated. The diagnosis is often missed, resulting in delayed management. Radiologists and clinicians should maintain a high index of suspicion for timely diagnosis and management. CRS plus IPEC is the standard of care for these patients and can significantly improve survival but is associated with considerable morbidity. Furthermore, it is crucial that the management of PMP be individualized because some patients may benefit more from debulking alone than from CRS combined with IPEC/systemic therapy or vice versa. Additional research into chemotherapy schedules and patient selection may shed light on other strategies to reduce morbidity and increase survival associated with this disease. More prospective trials should be conducted to formulate evidence-based guidelines for managing such patients.

## Data Availability

Not applicable.
